# Low Molecular Weight 35 kDa Hyaluronan Fragment HA35 Effectively Controls Post-surgery and Post-radiation Pain in a Patient With Hypopharyngeal Cancer: A Case Report

**DOI:** 10.7759/cureus.81957

**Published:** 2025-04-09

**Authors:** Zongchun Zhang, Xiaoxiao Jia, Dylan Treger, Hui Wang, Mizhou M Hui

**Affiliations:** 1 Department of Radiotherapy, Qingdao Cancer Hospital, Qingdao, CHN; 2 Hynaut Laboratories, Hynaut Group, Qingdao, CHN; 3 Miller School of Medicine, University of Miami, Florida, USA

**Keywords:** cancer pain management, hyaluronan, hypopharyngeal cancer pain, injection therapy, lower hyaluronan fragment ha35

## Abstract

Hypopharyngeal cancer, a rare form of throat cancer, is typically treated with a combination of surgery, chemotherapy, and radiation therapy. These treatments often result in severe pain and discomfort for patients, particularly after surgery and radiation therapy. Hyaluronan, a calcium channel TRPV1 blocker, can help alleviate pain. However, its clinical use is limited by its molecular weight, which affects tissue penetration. Low molecular weight hyaluronan fragments, such as HA35, have good tissue permeability and have been successfully used to treat various types of pain. We reported a case of a 50-year-old male patient with hypopharyngeal cancer, esophageal cancer, and metastases to the skull base. The patient reported nerve pain radiating to the temple due to surgical damage to the first cervical vertebra. This pain was unresponsive to five daily doses of painkillers following radiotherapy, and he required sedatives to sleep at night. He was treated with low molecular weight hyaluronan fragment HA35. The patient initially received a subcutaneous injection of 100 mg of HA35 in the shoulder and neck along with oral painkillers once daily. The timing of HA35 injections was based on the patient's self-reported numeric pain rating scale (NPRS), with an injection cycle typically lasting five to seven days per dose. After five injections, the patient experienced significant pain relief and no longer needed sedatives to sleep at night. Over a one-year follow-up period, the maintenance treatment with oral HA35 concentrate and the injection therapy following radiotherapy nearly controlled both regular pain and post-radiation pain. Moreover, the treatment substantially alleviated anxiety and fatigue while improving diet, sleep, and overall quality of life, as evidenced by improved scores on the European Organization for Research and Treatment of Cancer Quality of Life Questionnaire-Core 30 (EORTC QLQ-C30). No adverse reactions were observed throughout the treatment course. These findings not only support previous studies on the efficacy of hyaluronan in mitigating radiation-induced pain but also suggest that low molecular weight 35 kDa hyaluronan fragment HA35 may offer a promising, well-tolerated strategy for long-term cancer pain management and improved patient well-being.

## Introduction

Hypopharyngeal cancer, predominantly squamous cell carcinoma in 95% of cases, is a rare malignancy among head and neck cancers, accounting for only 3% of such cases. It is also the least well-managed within head and neck cancer treatment regimens [1]. Treatment typically involves a combination of surgery, chemotherapy, and radiation therapy [2]. These treatments often lead to severe pain, particularly post-surgery and post-radiation pain. Effective postoperative pain control and pain management are crucial for maintaining the quality of life and the physical and mental health of patients. However, there is limited research on effective pain control in patients with head and neck cancers, including laryngeal cancer [3]. As reported by Origill et al., only 35% of patients undergoing laryngectomy receive adequate and effective pain management [4]. Non-steroidal anti-inflammatory drugs (NSAIDs) have long been used for pain control across various diseases due to their anti-inflammatory properties [5,6]. Corticosteroids are also widely used as adjuvant therapy for cancer-related pain [7]. Opioids, although effective for producing long-lasting analgesia, are associated with adverse reactions such as nausea, vomiting, sedation, and impaired mobility, necessitating a reassessment of their use and management [8-10].

Low molecular weight hyaluronic acid fragment HA35, a type of hyaluronic acid found in human colostrum, is obtained through enzymatic digestion of high molecular weight hyaluronic acid by hyaluronidase from human colostrum or homologous sperm hyaluronidase [11,12]. Clinical reports suggest its efficacy in relieving inflammatory pain, neuropathic pain, and wound pain [13-15]. Research indicates that hyaluronic acid inhibits neurogenic inflammation and various acute or chronic pains by suppressing the activation of TRPV1 channels on nociceptors [16]. Herein, we present a case of laryngeal cancer pain successfully treated with HA35 injection, a novel analgesic agent.

## Case presentation

**Figure 1:**
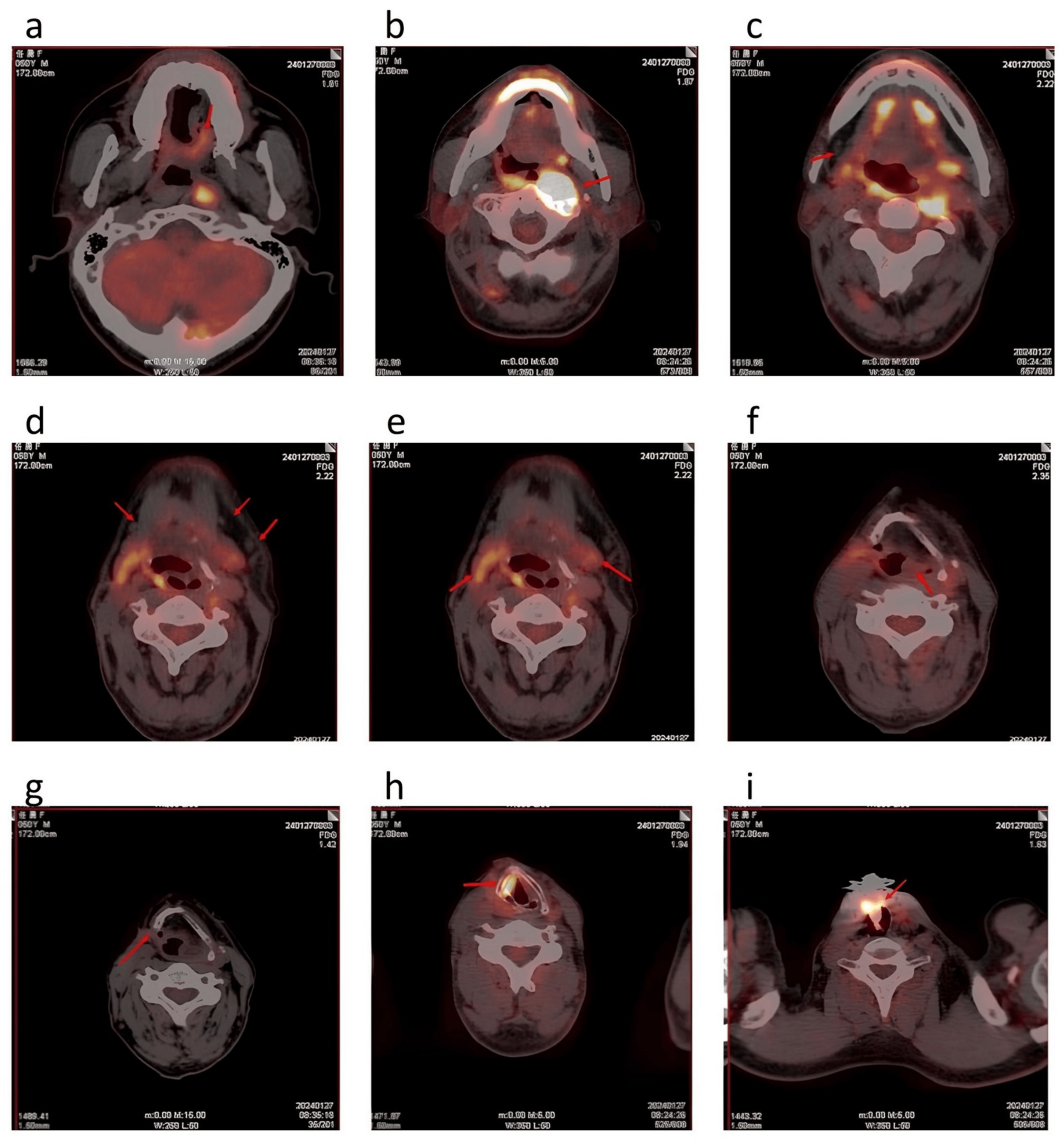
The patient's PET-CT scan images. 1a: After peripheral insertion of a central venous catheter (PICC), asymmetry of the bilateral pyriform sinuses was observed (red arrow). 1b: A soft tissue density lesion was visible in the left pyriform sinus region, with slightly increased radiotracer uptake (red arrow). 1c: No abnormal lymph nodes were observed in the right neck (red arrow). 1d: Multiple slightly enlarged lymph nodes were detected in bilateral level IB regions, with the largest having a short-axis diameter of approximately 6 mm and slightly increased radiotracer uptake (red arrows). 1e: A soft tissue mass was detected in the left parapharyngeal space and anterior to the cervical vertebra (red arrows), measuring 20 mm × 12 mm × 27 mm (transverse × anteroposterior × craniocaudal). 1f: Increased radiotracer uptake was noted in the muscle tissue surrounding the anterior aspect of the left atlas (C1 vertebra) (red arrow). 1g: Postoperative changes in the trachea were observed. Increased radiotracer uptake was seen in the soft tissue surrounding the anterior trachea (red arrow). 1h: Increased radiotracer uptake was found in the right laryngeal region (red arrow). 1i: Increased radiotracer uptake was detected in the soft palate (red arrow).

The patient is a 50-year-old male who initially presented to the hospital over 12 years ago with a complaint of hoarseness for two and a half months and hemoptysis. After undergoing a series of examinations, he was diagnosed with hypopharyngeal carcinoma and subsequently underwent surgical treatment, though specific details of the procedure were not available. Postoperative pathology confirmed squamous cell carcinoma, and no further adjuvant therapy was administered after surgery, as the tumor was deemed completely resected with clear margins and a low risk of regional lymph node metastasis, making additional treatment unlikely to provide significant benefit while increasing the risk of adverse effects. Three years ago, at the beginning of the year, the patient incidentally discovered a painless enlarged lymph node on the left side of his neck, which rapidly increased in size. A subsequent CT scan of the neck and chest revealed a mass lesion in the left pyriform sinus with enlarged lymph nodes in level III of the left neck. Laryngoscopic examination also indicated a mass in the left pyriform sinus. One month later, a repeat laryngoscopic examination showed a grayish cauliflower-like neoplasm in the left pyriform sinus. A fine-needle aspiration biopsy of the left cervical lymph node was performed, and pathological analysis confirmed moderately to highly differentiated squamous cell carcinoma with keratinization and necrosis. Based on the above findings, the patient underwent local radiotherapy with intensity-modulated radiation therapy (IMRT), receiving a total dose of 60 Gy in 30 fractions, in combination with systemic chemotherapy for six cycles using the TPF regimen (docetaxel, cisplatin, and fluorouracil). Concurrently, he received immunotherapy with camrelizumab at a dose of 200 mg every three weeks. After six months, camrelizumab monotherapy was continued at the same dose. Ten months later, PET-CT at our hospital, followed by endoscopic examination, revealed poorly differentiated squamous cell carcinoma in the middle esophagus. The patient subsequently underwent additional radiotherapy, consisting of 3D conformal radiotherapy (50 Gy in 25 fractions) and intracavitary brachytherapy (three sessions of 5 Gy each), along with chemotherapy using the PF regimen (cisplatin and fluorouracil). The following year, another PET-CT scan at our center (Figure 1) showed postoperative changes in the right hypopharynx, with structural disruption at the surgical site, partial resection of the hyoid and thyroid cartilage, and increased radiotracer uptake. Further evaluation identified a soft tissue mass in the left parapharyngeal space and anterior to the C1 vertebra, with bone cortex destruction and increased metabolic activity, suggesting new metastatic lesions. Consequently, the patient received another round of radiotherapy with stereotactic body radiation therapy (SBRT), delivering a total dose of 30 Gy in five fractions, along with chemotherapy using the nab-paclitaxel and carboplatin regimen.

Before this round of radiotherapy, the patient's hypopharyngeal and esophageal cancer had already metastasized to the skull base. After receiving radiotherapy, combined with previous surgical damage to the first cervical vertebra, the patient developed nerve pain near the temple and possible bone pain. This pain was managed with oral painkillers (compound aminopyrine phenacetin tablets, one tablet five times a day, approximately every three to four hours), but the effect was not significant, indirectly affecting sleep and requiring the use of diazepam (2 mg once daily) at night. Prolonged pain led to irritability.

After consultation with a specialist, it was determined that there is no relevant family history. The patient experiences strong adverse reactions to NSAIDs, corticosteroids, and opioids, including symptoms such as nausea, vomiting, dizziness, and gastrointestinal discomfort. These adverse reactions significantly limited the options for conventional pain management. Despite this, the patient remains mentally clear and can independently assess the intensity of pain. According to the Numeric Pain Rating Scale (NPRS), the self-reported pain score before treatment was 10, indicating severe pain that significantly affected daily life due to the throat cancer. Since being diagnosed, the patient has quit smoking and drinking. Given the severity of the patient's pain, we immediately prepared a local injection of 100 mg low molecular weight hyaluronic acid fragment HA35. This was obtained by mixing hyaluronidase injection (1,500 units per vial, approved by the China Food and Drug Administration, registration number H31022111, manufactured by Shanghai Shang Yao First Biochemical Pharmaceutical Co., Ltd.) and hyaluronic acid injection (10 mg/mL, approved by the China Food and Drug Administration, registration number H20174089, manufactured by Shanghai Haohai Biotechnology Co., Ltd.). The injection was administered subcutaneously in the patient's neck and shoulder area. We continuously monitor and observe the patient's pain relief and use the NPRS to allow the patient to self-assess the intensity of pain before and after treatment. The patient was still advised to take painkillers as prescribed.

As shown in Table 1, 20 minutes later, the patient reported an NPRS score decrease to 6, indicating a 40% relief. After 40 minutes, the score dropped to 3, signifying a 70% relief, with the best effect observed after 40 minutes. Six and a half hours after the first injection, the patient started taking oral painkillers (one compound aminopyrine phenacetin tablet) and did not use sedatives for sleep assistance that day. On days two to four, the patient took one painkiller tablet each day. The burning pain in the pharynx, temple, and trigeminal nerve was almost completely relieved, without affecting normal work and social activities. On the fifth day, the second injection was administered, followed by injections every seven days for a total of five injections.

**Table 1:** Pain scores and assessments at different time points in patients after first injection.

Treatment time	Pain Numeric Rating Scale (NPRS)	Pain relief percentage	Pain assessment
Before treatment	10 score	0%	Throat pain, temple pain, radiation-induced skin burns at the treatment site, and discomfort causing restlessness in the patient, requiring the use of sedatives for sleep aid at night.
Treatment for 20 minutes	6 score	40%	Pain relief of around 40%, with easing of throat and head pain starting.
Treatment for 40 minutes	3 score	70%	Overall pain reduction of approximately 70%, with the patient noticeably feeling relaxation in the head and able to move his head from side to side.
Treatment for 60 minutes	3 score	70%	Pain remains at its current state, with the patient noticeably feeling more energetic and willing to move around.
Treatment for 24h	3 score	70%	The patient's pain has not further decreased, but overall comfort is good, with a good appetite, and no need for sedatives for sleep aid.
Treatment for 5d	7 score	30%	Pain is starting to recur, but the intensity is not as severe as before treatment.

One month after the injection therapy, the patient's pain was well controlled. A follow-up was conducted for patients undergoing one-month injection therapy, with the patient's scores from the European Organization for Research and Treatment of Cancer Quality of Life Questionnaire-Core 30 (EORTC QLQ-C30), as presented in Table 2. Quality of life scores evaluated the effectiveness of drug therapy across six functional domains, an overall health composite domain, three symptom domains, and six other domains. In this case, the patient's scores for physical, role, emotional, cognitive, social functioning, and overall health status all showed improvement over the past week. Scores for fatigue, nausea and vomiting, pain, dyspnea, insomnia, loss of appetite, constipation, and diarrhea all showed a decrease or remained unchanged. Additionally, the HA35 injection did not cause any financial difficulties for the family and did not produce any side effects.

**Table 2:** Quality of life scores before and after patient treatment.

Index	Scores before treatment	Scores after 5 injections	Scores after 1 year
Physical function	73.3	93.3	100
Role function	66.7	100	100
Emotional function	41.7	91.7	93.3
Cognitive function	66.7	100	100
Social function	66.7	100	100
Overall health condition	41.7	75.0	91.7
Fatigue	44.4	22.2	0
Nausea and vomiting	33.3	0	0
Pain	100	16.7	0
Difficulty breathing	33.3	33.3	0
Insomnia	100	0	0
Loss of appetite	66.7	0	0
Constipation	0	0	0
Diarrhea	33.3	0	0
Economic hardship	0	0	0

A one-year follow-up was conducted for the patient. During subsequent treatment, the patient reported primarily maintaining therapy with an oral solution of HA35 concentrate (5,000 mg/50 mL, Qingdao Huinorde Biotechnology Co., Ltd.), taken once daily or split into two doses per day, with each treatment cycle lasting 21 days. After more than six months of adherence to this regimen, the patient exhibited good mental and physical condition, and the tumor remained stable after radiotherapy without further expansion. Consequently, the doctor strongly recommended another round of radiotherapy. However, following the radiotherapy, the patient experienced radiation-related pain again, which was managed with repeated HA35 injections. The patient reported that, after receiving five injections, the pain in the larynx, pharynx, and temple caused by radiotherapy was successfully alleviated. According to the scores presented in Table 2, one year after treatment, the patient's quality of life and physical condition had recovered to nearly normal levels.

## Discussion

The findings of this study indicate that targeted subcutaneous administration of HA35 in the shoulder and neck region appeared to reduce pain levels among individuals undergoing postoperative and post-radiotherapy interventions for hypopharyngeal carcinoma. Importantly, these findings also suggest HA35 as a therapeutic analgesic, evidenced by its gradual reduction in medication dosage post-treatment and alleviation of other post-treatment symptoms, ultimately leading to improved quality of life scores (Table 2). As previously discussed, the current treatment modalities for hypopharyngeal cancer lack well-developed pain management mechanisms, leaving patients with inadequate relief from symptoms such as throat pain, ear pain, pain upon swallowing, and throat discomfort from coughing while drinking [4]. In comparison to commonly used medications for managing postoperative and post-radiotherapy pain in hypopharyngeal cancer patients, such as non-steroidal anti-inflammatory drugs, corticosteroids, and opioids, HA35 demonstrates fewer adverse effects in treating bone metastasis-related pain. To our knowledge, this case report presents the initial successful management of hypopharyngeal carcinoma pain using HA35.

Hyaluronic acid and its fragments are abundant in the naked mole-rat, known for its lifelong cancer immunity and relatively insignificant pain from inflammation [17]. In lymphatic imaging studies using 125I-HA35, effective dermal penetration and superior tissue permeability of the HA35 fragment were observed, promoting the homing of macrophages and immune cells to lymph nodes and exerting anti-inflammatory effects [12]. The efficacy and safety of HA35 in pain management have been demonstrated in several high-quality clinical trials with adequate sample sizes [13-15]. Therefore, our report underscores HA35 as effective and safe for managing pain associated with hypopharyngeal cancer.

While there is no direct evidence indicating that hyaluronic acid alleviates pain specifically related to hypopharyngeal cancer, studies suggest its potential in mitigating side effects of radiation therapy, including skin pain and pain frequency [18]. A prospective randomized clinical study has indicated its efficacy in alleviating acute radiation dermatitis pain caused by radiotherapy in patients with head and neck squamous cell carcinoma [19]. Upon further follow-up with continuous injections for the patient, it was found that, under the regimen of one injection of HA35 solution every seven days and the use of HA35 injections after repeated radiotherapy, the patient no longer required pain medication. Additionally, with the continued oral administration of HA35 concentrate and repeated post-radiotherapy injections, the patient demonstrated significant improvement in overall well-being and physical function, reaching levels comparable to those of a healthy individual. Therefore, our study not only corroborates the above findings but also addresses the gap in hyaluronic acid treatment for cancer pain, offering broad clinical significance and utility.

Limitations of this report include the sole reliance on pain scoring scales to present pain intensity, which may lack objectivity. To provide a more comprehensive understanding, we supplemented these scales with quality of life assessments using the EORTC QLQ-C30, allowing for a wider capture of symptoms and events and enabling patients to describe their experiences more comprehensively [20].

## Conclusions

Subcutaneous injection of low molecular weight 35 kDa hyaluronan fragment HA35 effectively controlled post-surgery and post-radiation pain in a patient with hypopharyngeal cancer. It also alleviated the patient's anxiety and fatigue symptoms and improved diet and sleep quality. Additionally, during the maintenance phase after pain relief, oral HA35 further improved the patient's mental state and overall well-being. Therefore, we believe that low molecular weight 35 kDa hyaluronan fragment HA35 can be clinically promoted and applied as a cancer pain management strategy.
